# Innervation pattern and fiber counts of the human dorsal nerve of clitoris

**DOI:** 10.1038/s41598-024-72898-8

**Published:** 2024-10-04

**Authors:** Elçin Tunçkol, Christine Heim, Irene Brunk, Imre Vida, Michael Brecht

**Affiliations:** 1 Bernstein Center for Computational Neuroscience Berlin, Humboldt-Universität zu Berlin, Philippstrasse 13, Haus 6, Berlin, Germany; 2 Institute of Medical Psychology, Charité–Universitätsmedizin Berlin, Corporate member of Freie Universität Berlin and Humboldt-Universität zu Berlin, 10117 Berlin, Germany; 3 Institute for Integrative Neuroanatomy, Charité - Universitätsmedizin Berlin, CCM, Philippstrasse 12, 10115 Berlin, Germany; 4 NeuroCure Cluster of Excellence, Humboldt-Universität zu Berlin, Berlin, Germany

**Keywords:** Anatomy, Urology, Somatic system

## Abstract

Even tough clitoris plays a critical role in female sexuality, we lack a precise understanding of qualitative and quantitative aspects of the innervation of the human clitoris. To address this issue, we dissected human clitorides from body donors and imaged them after staining with iodine with microCT for a macroscopic analysis. To resolve innervation patterns at the microscopic level we prepared thin sections of clitorides and stained them with trichrome azan to reveal the tissue structure combined with immunocytochemistry against Neurofilament H antibodies to reveal all axons and luxol blue labeling to reveal myelinated axons. We find the clitoral branch of pudendal nerve that innervates the clitoris not as single nerve, but as number of loose bundles. In the crus of the clitoris, about 12 such bundles can be recognized while about 32 bundles are present in the clitoral hemi-body. We counted on avarage 2917 axons in the crus of the clitoris (76% of which are myelinated) and 3137 axons in the hemibody of the clitoris (71% of which are myelinated). While the human clitoris receives only one third of the number of axons that innervate the human penis, an estimate of innervation density (per surface area) revealed that clitoris has approximately 6 times denser innervation compared to the penis. Thus, our study combines histology with microCT analysis provides detailed information on the number, myelination and innervation density of dorsal nerve of clitoris.

**Supplementary Information:**

The online version contains supplementary material available at 10.1038/s41598-024-72898-8.

## Introduction

For centuries the human clitoris has not been a focus of scientific investigation even though its important role in female sexuality was known since the dawn of the time. In the 16th century this systematical neglect was ceased by the curiosity of renaissance physicians^1^, which was followed by Kobelt’s detailed anatomical studies on male and female genital organs^2^. However anatomical recognition was not sufficient to eradicate the shame that was built around the female genitalia. Consequently, the clitoris was anatomically reduced to only its visible tip, the glans, and seen as defective and inferior compared to its male counterpart. This “underdeveloped penis” approach determined the *Zeitgeist* about theories on female sexuality and pleasure. Freud suggested that clitoris is the immature pleasure zone and it should be replaced by vagina as the female child matures into a “real woman”^3^. This biased idea obscured rather than clarified female sexuality. As a result, some women were subjected to surgical procedures to alter the position of their clitoris, while others were involuntarily castrated^3,4^.

This artificial dichotomy of vaginal versus clitoral orgasm was first challenged by the revolutionary studies of Masters and Johnson in which the contribution of every element of vulva in female sexual response was documented^5^. Later studies demonstrate the close anatomical and functional relationship between clitoris, vagina and urethra^6–8^. Since these structures not only move in a coordinated fashion during sexual activity but also they share vasculature and innervation^9^, it was suggested to be renamed as the clitourethrovaginal (CUV) complex^10^.

One of the components of CUV complex is the female corpus spongiosum. The male and female corpus spongiosum share the same embryological origin. The male corpus spongiosum supports the penile urethra and forms the glans penis; while female corpus spongiosum develops separate from urethra and makes up the vestibular bulbs^11^. There has been a debate about if the female corpus spongiosum is the distal extension of vestibular bulbs^12^ or a different kind of tissue, namely pars intermedia^13,14^. However, histological data remains inconclusive and a modern approach is required for this debate.

This complex network of erectile tissues is heavily innervated by somatosensory afferents and autonomic fibers. The main afferent fibers belong to the dorsal nerve of the clitoris (DNC), which is the branch of pudendal nerve travelling along the ischiopubic ramus of the pelvis and innervating the glans clitoris^15,16^. Previous studies suggested that DNC does not ramify much along the clitoris^17^, concentrated on the dorsal half of the clitoral body but innervate evenly the glans clitoris^18^.

This well-known “seat of pleasure” still holds mysteries. Even tough in the last decade our anatomical knowledge on clitoris has increased greatly, our lack of understanding between anatomical compartments and their representation in the nervous system stems from the scarcity of data. Therefore, we try to answer the following questions with the current study: (i) What is the relationship of the human female corpus spongiosum to the clitoris (ii) where are the bundles of dorsal nerve of clitoris located and how do they ramify? (iii) how many fibers are there in different levels of dorsal nerve of clitoris and (iv) what is the myelination fraction of fibers?

## Materials and methods

### Clitoral tissue preparation

Formalin-fixed vulva samples were collected from 6 female cadavers from the body donation program of the Center for Anatomy, Charité Universitätsmedizin Berlin. The donors have given informed written consent for the use of their bodies for educational and research purposes before they had deceased. All the related documents are stored in the Prosectur of the Center of Anatomy, Charité Universitätsmedizin Berlin. All procedures were carried out in accordance with the “Law for the regulation of the anatomical dissection” (Sektionsgesetz, SRegG BE)^19^ and the “Law on body and burial services” (Bestattungsgesetz, BestattG BE) of the federal state of Berlin, Germany^20^ as well as the ethical standards and the declaration of Helsinki 1964 and its subsequent updates. According to German law, no further ethical approval from institutes or commissions was required because the tissues were collected from cadavers as explained above. Personal information of donors was confidential and was unknown to the researchers except for their age. The age range of donors was 75–98 years and the mean age was 88 years. After further dissection, samples were preserved in 70% alcohol for two days. Subsequently, the tissue was embedded in paraffin as previously described^21^. Paraffin blocks were cut into 7 μm sections on Leica Jung RM2035 rotary microtome (Leica Instruments GmbH, Germany, Cat# 042819658) and mounted on Thermo Scientific Superfrost Ultra Plus^®^ Gold slides. Consecutive slides were prepared for immunohistochemistry, Luxol fast blue and trichrome Azan staining.

### Immunohistochemical staining

Immunohistological procedure was carried out as previously described^21^. Clitoris tissues were deparaffinized and treated with citrate buffer (Antigen Unmasking Solution, Citric Acid Based, pH 6, 100× concentrated stock solution, Vector Laboratories Cat# H-3301, RRID: AB_2336227 for heat-induced epitope retrieval (HIER), blocking solution and primary antibodies against Neurofilament H (Chicken polyclonal, Millipore Cat# AB5539, RRID: AB_11212161) sequentially. Fourty-eight hours later, sections were incubated with secondary antibodies, coupled to the fluorophore Alexa 488 (Thermo Fisher Scientific Cat# A-11039, RRID: AB_2534096). Finally, the sections were mounted on glass slides with Fluoromount G^®^ mounting medium (Biozol, Eching, Germany, Cat# Nr.: SBA-0100-35) and cover-slipped.

### Luxol fast blue staining

In order to visualize myelinated fibers, we used the Luxol fast blue stain as previously described^21^. After deparaffinization, slides were treated with 1% solvent Blue 38 (Sigma, Cat# S3382) dissolved in 96% ethanol and acetic acid at 56˚C overnight. The slides were rinsed and differentiated using lithium carbonate (American MasterTech Scientific, Cat# KC2622) and 70% ethanol. The tissue was cleared with 100% ethanol and Xylol. Sections were mounted with Eukitt^®^ (Sigma- Aldrich, Cat#03989).

### Trichrome azan staining

After deparaffinization Heidenhain`s azan trichrome staining protocol was followed. Sections were subjected to azocarmine (Morphisto, Cat#10147), aniline ethanol (Morphisto, Cat#10138), acetic acid in ethanol (Morphisto, Cat#11374), phosphotungstic acid (Morphisto, Cat#10324), and anilin blue- orange G (Morphisto, Cat#10144), consecutively. Finally, sections were cleared with Xylol and Mounted using Eukitt^®^ (Sigma- Aldrich, Cat#03989) (Supplementary Table 1).

### Microscopy

Z-stack images were obtained using a Leica DM5500B epifluorescence microscope with a ×63 oil objective (axial resolution 0.772 μm) with 1-µm axial steps. The images obtained were from 1600 × 1200 to 8696 × 7706 pixels in size with a field of view between 203 × 152 μm and 1103 × 977 μm. Image stacks were analyzed using the Fiji version of ImageJ (RRID: SCR_002285)^21^.

### MicroCT imaging

We used diffusible iodine-based contrast enhanced computed tomography (diceCT) to analyze and visualize penile structure and nerves as previously described^21^. To enhance the contrast for microCT a whole clitoris without the connective tissue around was incubated in 1% iodine solution for 1 week. Computed tomography scans were obtained by YXLON FF20 CT system (YXLON International GmbH, Hamburg, Germany; RRID: SCR_020903). Scans were performed with an isotropic voxel size of 10.6 μm. Images were visualized and segmented using an extended version of the Amira software (AmiraZIBEdition 2022, Zuse Institute Berlin, Germany). Segmentation was done manually with a combination of the ‘Threshold’, ‘Brush’ and ‘Lasso’ module^21^.

### Quantification of the nerve fibers and statistical analysis

Quantification of pudendal fibers was done at the middle of crura for both sides of the specimen. The DNC bundles in the body of the clitoris do not have clear hemispheric boundaries. Therefore bundles were assigned one of the hemibodies according to their position relative to the deep dorsal vein. NFH and LFB positive fibers were counted manually on one slide per each. The details of methodology are provided in Supplementary Fig. 1. We used SPSS Statistics version 29 (IBM, New York, USA; RRID: SCR_002865) for statistical analysis. The normality of the data was tested with one sample Kolmogorov- Smirnov test and the properties of distributions were described. The difference between the nerve density of penis and clitoris was tested with Mann- Whitney U test.

### Comparison of nerve density in penis and clitoris

In addition, we wanted to compare the innervation density of penes and clitorides. In a previous study we counted the number of fibers innervating the penis^21^ and for density comparison we used penes that were counted previously. In order to calculate surface density, the lengths of clitoris and penis samples from the pubic arch to the tip of glans were measured. Circumference was measured at the proximal shaft, middle of the shaft and proximal to the corona of glans and averaged for 3 penes. Due to connective tissue around the clitoris circumference was calculated by estimating the diameter corresponding slices of 3 clitorides. Lastly, both clitoris and penis were considered as cylindrical structures and their surface area estimated accordingly.

## Results

### Anatomy of vulva and clitoris

**Fig. 1:**
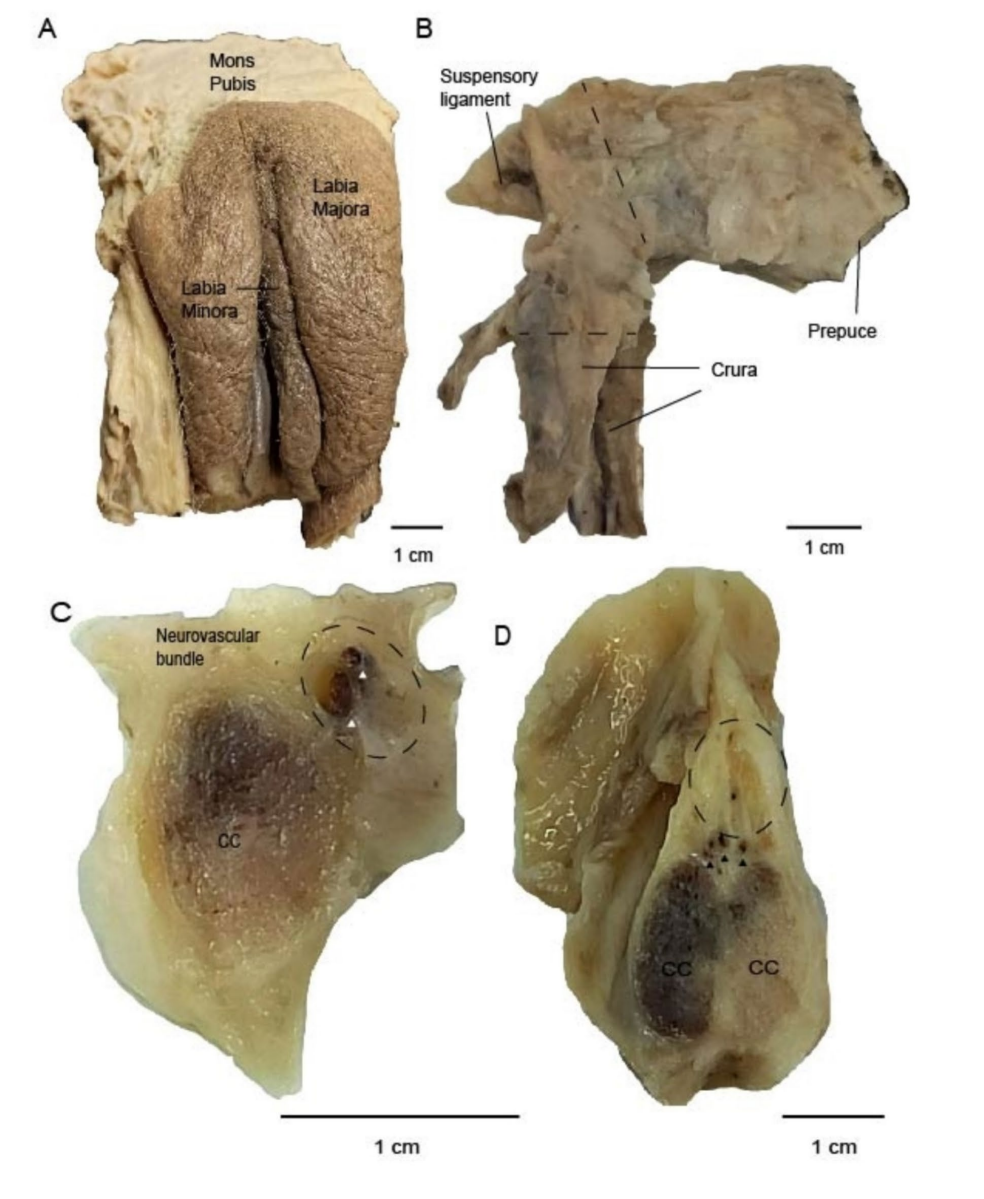
External structure of human vulva and the sectioning of the clitoris. (**a**) Female vulva in frontal view. (**b**) The body and the crura of clitoris are shown in lateral view. Dashed lines show where we took samples. (**c**) Cross section of the crus of clitoris shows the corpus cavernosum (CC) and neurovascular bundle. Arrows show blood vessels accompanying pudendal bundles. (**d**) Cross section of the clitoris at the level of pubic symphysis. Dorsal nerve bundles of clitoris are situated dorsal to erectile bodies. Arrows show blood vessels.

The borders of vulva specimens were anteriorly the mons pubis, posteriorly perineum and laterally the medial surfaces of thighs. Between the mons pubis and perineum the labia majora are situated laterally. Between labia majora, one observes the labia minora, skin-folds covering connective and vascular erectile tissue. The lower part of the bilateral labia minora converge in the midline inferior to clitoris and forming the frenulum; while the upper parts form the prepuce (Fig. 1A).

We further dissected the skin and the connective tissue around the clitorides until the body of the clitoris and bilateral crura were exposed. Crura travel alongside the ischiopubic rami until the pubic symphysis where they meet in the midline and form the clitoral body. Suspensory ligament keeps the clitoris attached to pubic symphysis and preserves its shape (Fig. 1B). The crura are composed of corpus cavernosum, neurovascular bundle and connective tissue. A cross-section of the crus shows the neurovascular bundle that was attached to ischiopubic bone laterally. Neurovascular bundles consisted of blood vessels and branches of pudendal nerve coming towards clitoral body (Fig. 1C). Bilaterally they make an arch inferior to pubic symphysis. Branches of pudendal nerve enter the clitoral shaft mediodorsally and spread between 11 and 1 o’clock direction; forming the dorsal nerve of clitoris (Fig. 1D). Our data provide an overview of clitoris anatomy and the neurovascular bundles innervating this structure.

### MicroCT scanning and corpus spongiosum: pars intermedia

**Fig. 2:**
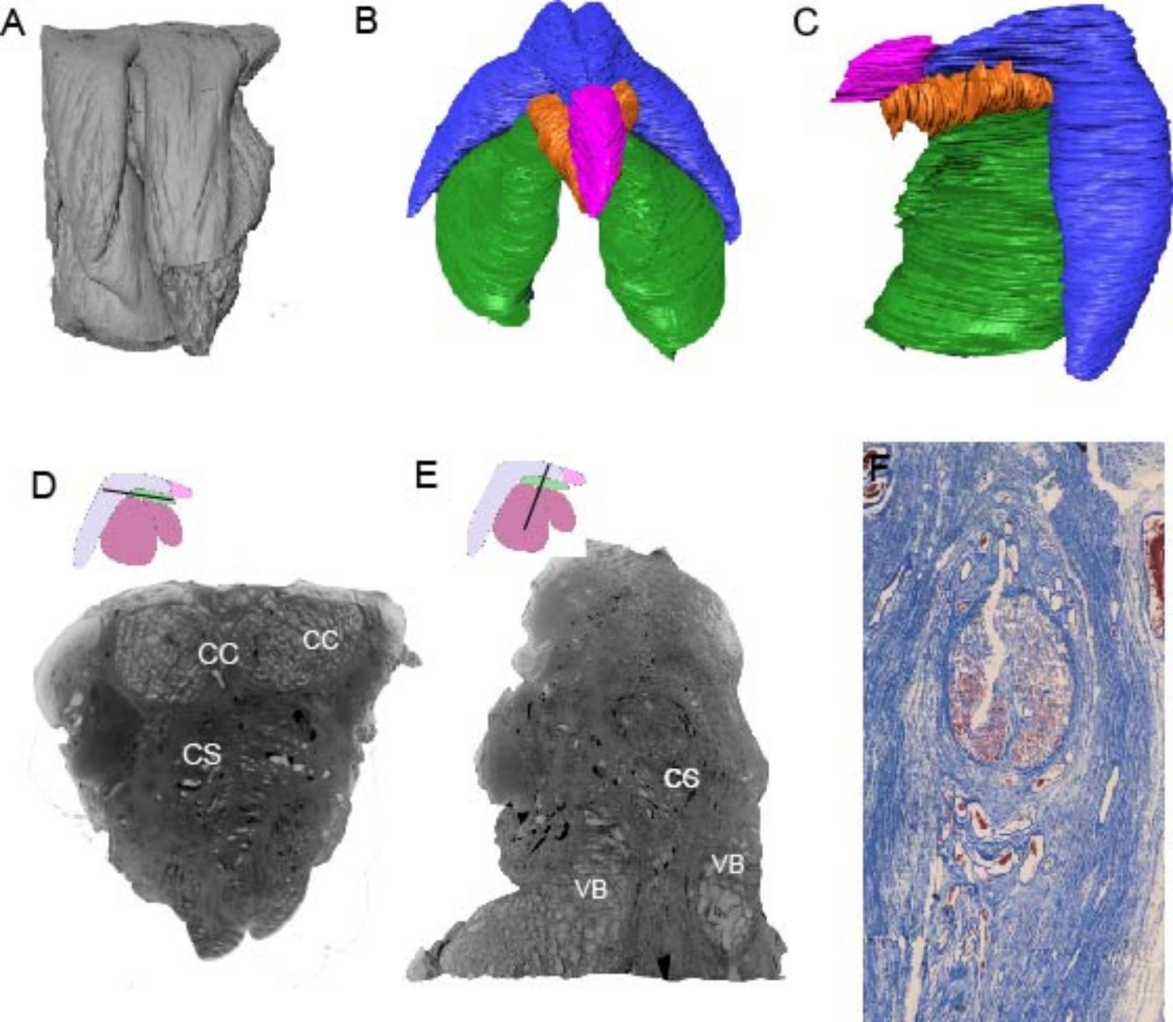
MicroCT scan of the human clitoris and female corpus spongiosum. (**a**) Another vulva is microCT scanned shown in frontal view. (**b**) Volume rendering of segmented structures of clitoral structures. Corpora Cavernosa (CC), corpus spongiosum (CS), vestibular bulbs (VB), and glans clitoris (GC) are represented in blue, orange, green and pink, respectively. (**c**) Volume rendering of segmented structures in lateral view. (**d**) Transverse section of clitoris showing different type of erectile tissues. (**e**) Cross-section of clitoris showing the same erectile tissue. (**f**) Cross-section of another clitoris that was stained with trichrome azan showing corpus spongiosum in relation to corpora cavernosa.

The external structure of iodine treated vulva is shown; labia majora, labia minora are recognized (Fig. 2A). The volume rendering output of segmented clitoral structures are also shown (Fig. 2B). Corpora cavernosa, forming the shaft and the glans of the clitoris were identified. Between the two crura, the vestibular bulbs and, anteriorly, in the midline, a triangle shaped erectile tissue were observed (Fig. 2C). This triangular erectile tissue was located inferior to clitoral body and superior to vestibular bulbs. Even tough it had a close connection with vestibular bulbs and clitoral body, it had more spacious trabeculae compared to them and was surrounded by non-erectile tissue. As seen in the volume rendering this tissue had less clear boundaries than vestibular bulbs and clitoral body and extending towards to the glans clitoris (Fig. 2D,E). In another specimen, trichrome azan staining was used to confirm the structural differences that were observed in the scans. Irregular and large spaces of pars intermedia were observed inferior to clitoral body (Fig. 2F). MicroCT scans and trichrome azan staining provide an overview of inner anatomy of the clitoris.

### Trajectory of dorsal nerve of clitoris

**Fig. 3:**
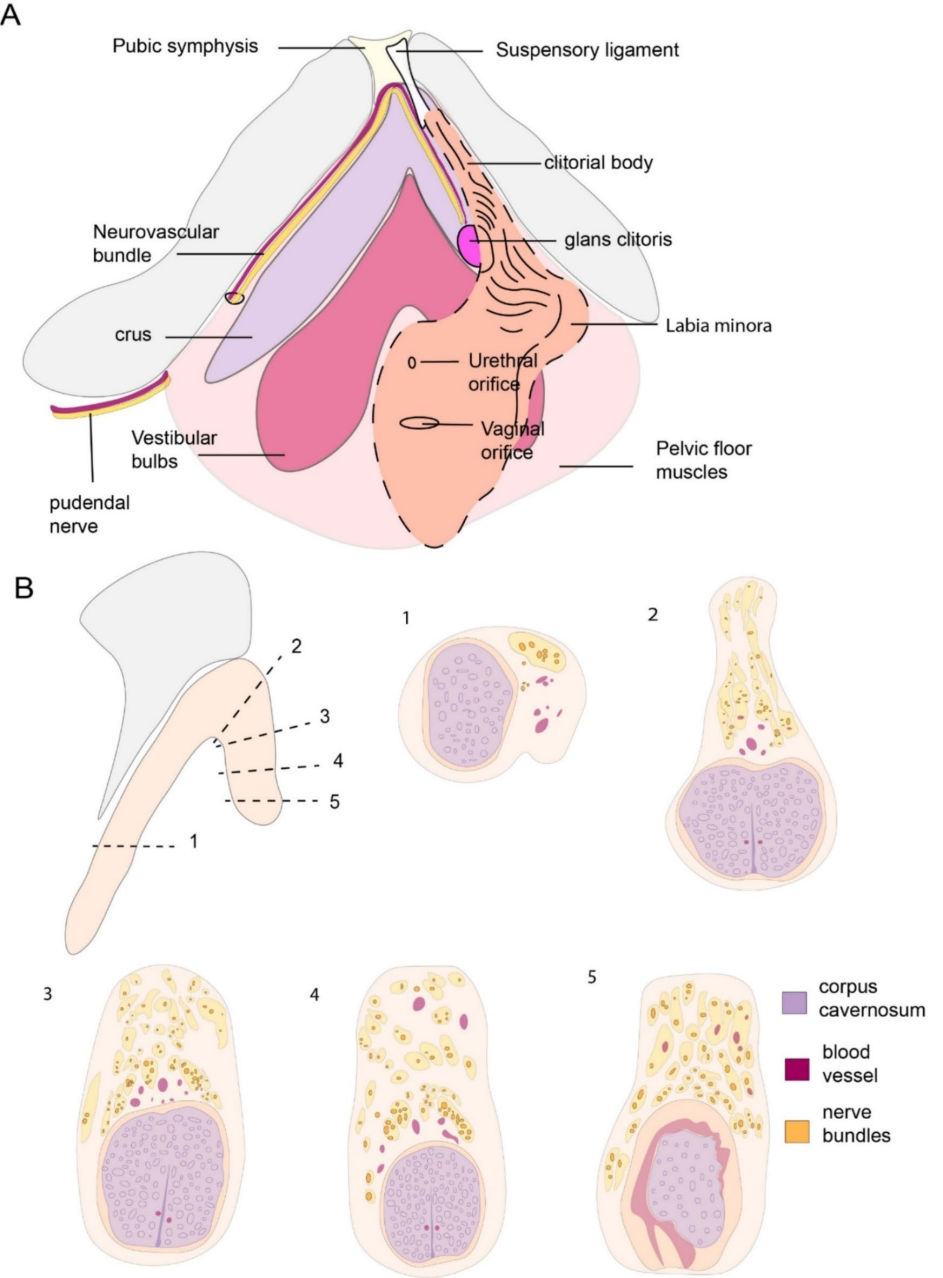
Distribution of bundles of dorsal nerve of clitoris. (**a**) Drawing shows the pelvic structures. On the left side of the drawing, the trajectory of pudendal is shown. Neurovascular bundle is situated between corpus cavernosum and ischiopubic ramus. On the right side, labia minora and external skin folds are shown with respect to clitoris. (**b**) Simplified clitoris is shown in lateral view. Numbers with dashed lines demonstrate the sections that were used for drawings. (1) Transverse section of crus showing the neurovascular bundle that is situated between corpus cavernosum and iscihopubic ramus. (2) Cross section of the body of clitoris from the pubic symphysis. Bilateral bundles and corpora cavernosa join together. (3) Cross section of the proximal body of the clitoris. Bundles are distributed in dorsally. (4) Cross section of distal body of the clitoris. Bundles are distributed more sparsely. (5) Cross section prior to glans clitoris. Bundles are distributed dorsally and dorsolaterally.

The pudendal nerve branches out after leaving the pudendal canal. The pudendal branch that innervates the clitoris follows ischiopubic ramus along crura and makes an arch dorsally to enter the body of clitoris dorsally under the symphysis (Fig. 3A). The distribution of the nerve bundles changes along the trajectory (Fig. 3B). At the level of crura (Fig. 3B.1), bundles were positioned between the bone and the crus. Bundles are bigger and close to each other proximally, reflecting how the branches gradually fan out. At the shaft of the clitoris (Fig. 3B.2) where the bilateral DNC converge inferior to pubic symphysis, bundles were distributed dorsally and dorsomedially (between 11 − 1 o’clock). Throughout the body of the clitoris bundles spread dorsolaterally (between 10 − 2 o’clock) and in a larger area (Fig. 3B. 3–4). The distance between bundles were increased and individual bundles got smaller. Before innervating the glans and prepuce some bundles were observed in laterally as well (Fig. 3B.5). Thus, our sections provide a detailed of the description of the trajectory of the dorsal nerve of the clitoris.

### Quantification of nerve fibers along the crura

**Fig. 4:**
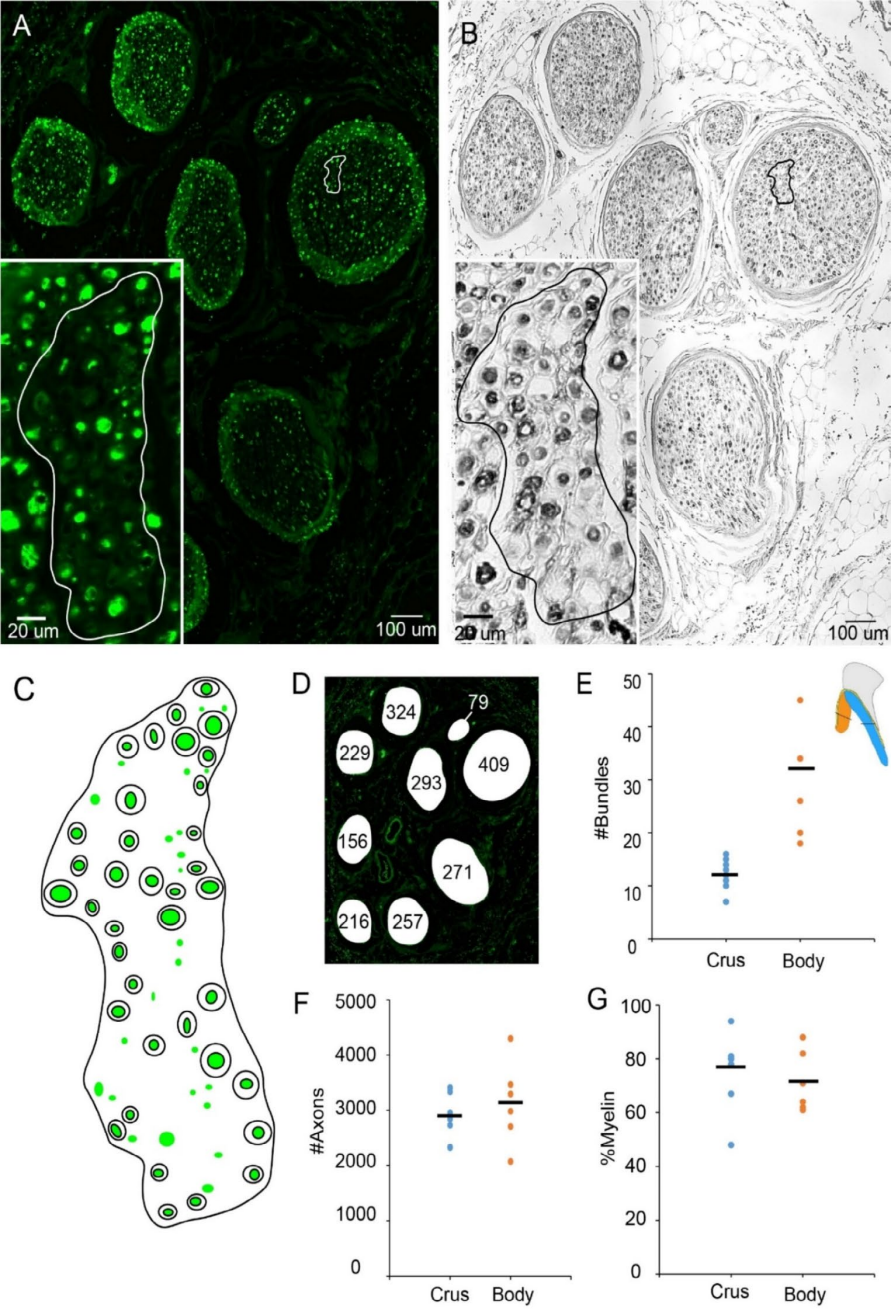
Nerve fiber counts and myelination of human dorsal clitoral nerve at the level of crura and the middle of clitoral body. (**a**) Nerve bundles of crura stained with anti-neurofilament-H antibody (green fluorescence), a pan-neuronal stain for peripheral sensory axons. Inset shows the highlighted area in one of the bundles. (**b**) Same bundles in an adjacent 10-µm section were stained with Luxol fast blue to reveal myelin sheath. The inset shows the same area as in panel (A) (**c**) Schematic of myelinated and unmyelinated fibers seen in A and (B) Green marks represent the NF-H positive fibers while doughnut shapes represent myelinated fibers in the highlighted area. (**d**) The number of individual axons in each bundle is shown schematically. (**e**) Plot of the number of bundles for 8 crura and 6 hemi- clitorises. On the right corner drawing shows a reference clitoris which matching the colors of data points. The lines show the mean number of bundles. (**f**) Plot of the total number of axons bundles for crura (*n* = 8) and mid- body (*n* = 6) sections of clitorises. The lines show the mean axon count. (**g**) Plot of the fraction of myelinated fibers in bundles for 8 crura and 6 hemi- clitorises. The lines show the mean myelination fraction.

Consecutive sections were stained using a neurofilament H antibody and luxol fast blue are shown, respectively (Fig. 4A,B). The same area of the selected bundle was detected on consecutive slides and an overlay of the fibers was drawn (Fig. 4C). The big nerve bundles were found to have more fibers than small ones (Fig. 4D). In the crura bundles are organized in close proximity. Our data from 8 crura from 4 female subjects showed that there are 12 ± 3 bundles on average (mean ± SD). (Fig. 4E). The number of individual nerve fibers was found to be 2917 ± 446 (mean ± SD). (Fig. 4F). One sample Kolmogorov- Smirnov test showed that both number of bundles and fibers were normally distributed. Finally, the fraction of myelinated fibers was found to be 76% (Fig. 4G).

### Quantification of dorsal nerve fibers

In 4 specimens, 6 clitoral hemibodies were collected and fibers of DNC were counted. Bundles were scattered on the dorsomedial and dorsolateral aspect of corpus cavernosum and they were encapsulated by connective tissue. There were bundles 32 ± 13 (mean ± SD) in hemi-clitorises (Fig. 4E) and the contained 3137 ± 753 individual fibers on average (Fig. 4F). One sample Kolmogorov- Smirnov test showed that both number of bundles and fibers were normally distributed. Finally, the fraction of myelination was found to be 71% (Fig. 4G).

### Comparison of nerve density in clitoris and penis

The mean length of clitoris was 34 mm long with a mean circumference of 39 mm. The mean penile shaft length was 120 mm with an average circumference of 104 mm. Descriptive data is shown in Supplementary Table 2. Taking the number of fibers in the crura and the root of the penis and found to be 4 and 0.7 mm^2^, respectively. Our estimates indicate the clitoris has 6 times denser innervation than the penis. A Mann- Whitney U test showed that this difference was statistically significant (*p* = 0.05).

## Discussion

We studied the distribution pattern and fiber composition of the dorsal nerve of the clitoris in 5 females. Moreover, we microCT scanned one vulva to visualize the how it composed of different type of erectile tissue. We found that the neurovascular bundle in unilateral crus contains 12 bundles on average with different sizes and these bundles contain 2917 fibers on average. 76% of fibers in these bundles were found to be myelinated. In the middle portion of the clitoral body there are 32 bundles and with 3137 fibers unilaterally. 71% of these fibers were myelinated.

The approach of our study – the combination of fiber counts and microCT imaging – is novel in research on the clitoris. This is the first study that not only focuses on pudendal branch innervating clitoris and the dorsal nerve of the clitoris separately but also gives a comprehensive overview of nerve fibers throughout the whole clitoris. In addition, we quantified the extent of myelination of fibers innervating the clitoris and we determined ramification patterns of bundles of the dorsal clitoral nerve.

Despite the close topographical relationship and synergistic work between the elements of CUV complex, we do not have the full grasp of the structure and function of the erectile bodies in healthy sexual functioning and orgasm. The female corpus spongiosum- pars intermedia is one of the poorly defined structures. MicroCT scans showed clearly a different type of erectile tissue than vestibular bulbs and corpora cavernosa. It is situated in the midline, inferior to clitoral body extending towards glans clitoris. These results are in line with previous research describing histological differences between pars intermedia and corpus spongiosum of vestibular bulbs^13,14^. Even though anatomical structures are still in debate, it is known that all of the elements of CUV complex have increased vascular supply during the arousal^22,23^. Since glans clitoris has no erectile properties itself as in the penis, pars intermedia might provide stability to glans clitoris during intercourse. Another possibility is that it might act like a bridging structure between clitoris and urethra. The urethra is not only surrounded by erectile tissue itself^24^ but also anatomically packed together with distal vaginal wall and vestibular bulbs^6^. It has been observed that during orgasm it engorges^5^ and contributes to female ejaculation as an orgasmic expression^25^. Female ejaculation is the secretion of a milky fluid in small amounts from Skene’s glands. Skene’s gland is paraurethral gland that also known as the female prostrate because of the presence of prostate specific antigen (PSA). Another phenomenon that is observed in response to high levels of mechanical stimulation is the gushing of clear fluid from urethra, namely squirting^26^. Squirting is different from female ejaculation in color, volume and the constituents. The former is a form of diluted urine containing urea, uric acid, creatinine and PSA while the latter only PSA, glucose and fructose^27^. Even tough these orgasmic expressions are rare; they should not be confused with urinary incontinence (UI). Women describe ejaculation and squirting experiences as pleasurable and enriching^28^ while urinary incontinence during intercourse is a result of previously diagnosed physiological problem^29^. For instance, women who have been diagnosed with stress UI experience involuntary micturition during penetration because of dysfunction of sphincter muscle; while ones with urgency UI experience involuntary micturition during orgasm because of over active detrusor muscle^29^. Considering the shared vascular and nerve supply of CUV complex^30^ as well as the synergetic activation during orgasm more research needed to comprehend female orgasm and its diverse expressions.

Furthermore, clitoris is an organ with considerable amount of individual variance in morphology and histology^31^. We are still far from understanding the factors contributing to this variety and how it affects the female sexual functioning. Likewise, we can only speculate about a relationship between the size of the organ and the number of fibers innervating it. Clearly more research is needed not only to understand the influence of genetic and environmental factors on the anatomical and neural connections of the clitoris, but also the contribution of these factors in healthy female sexual functioning.

In a previous study, we found that there are approximately 4400 fibers unilaterally in the root of penis and 60% of these fibers were myelinated^21^. In contrast, in crus of clitoris we counted on average 2917 fibers and 76% of these fibers were myelinated. Thus, the clitoris has a lower fiber count but the proportion of myelinated axons is higher. Moreover, when we consider the size and surface area of these two phallic organs, the innervation density of clitoris is approximately 6 fold higher than in the penis.

In the proximal shaft of penis, there were on average 8300 fibers unilaterally and 45% of them were myelinated, while in the middle of the clitoral body we counted on average 3137 fibers unilaterally and 71% of them were myelinated. Comparison of the fiber numbers in the root and shaft of these phallic organs suggests that there might be a branching of nerve fibers along the nerve in both penis and clitoris in addition to anastomoses with autonomic fibers. The degree of branching seems to be substantially higher in the dorsal nerve of penis. This difference might be a consequence of the size difference between penis and clitoris. In the penis the distance between the shaft and glans penis is longer, so fibers need to travel longer distances to reach to the glans. While traveling to glans they ramify along the penis, increasing the fiber counts^21^ before innervating the skin as free nerve endings^32^. In the case of clitoris, the distance between shaft and glans is shorter, so bundles travel with less ramification without losing myelination. Moreover, previous research showed that a specialized type of mechanoreceptor named genital end bulb was more abundant in glans clitoris compare to glans penis^33^. Clearly the myelination is more important for female sexual functioning. A pioneering study, which described the mechanosensory properties of these end organs in mice and found that those low threshold corpuscles are innervated by rapid adapting fibers and tuned to light touch and mechanical vibrations. Finally, the same authors found that these corpuscles are more abundant in glans clitoris than glans penis and were crucial for sexual function^34^. Further studies are required to reveal the function and importance of myelin in female sexual functioning.

Previous research showed that dorsal nerve of clitoris composed of autonomic fibers as well as sensory fibers^17^. A recent study estimated 5100 fibers unilaterally in the proximal clitoris, at the level of the pubic symphysis^35^. Despite the fact that their estimation is higher than our counts, differences in sampling methods and subject profile should not be neglected. In our study we had access to tissue from elderly women, while in the other study the tissue from younger adults who received hormone therapy before the gender affirming surgery was used^35^. Therefore one possible reason for the difference in fiber counts might be the age of the subjects. With the onset of menopause female genitalia undergoes a series of changes. Decrease in elasticity and lubrication of vagina, loss of vascularization in labia majora^5^, gradual increase in vibratory threshold of clitoris^36^ are some of them. Considering the loss of myelinated and unmyelinated fiber as well as axonal atrophy in the peripheral nervous system^37^, we can assume that there was loss of fibers not only in the dorsal nerve of clitoris but also in pudendal nerve. Moreover, at the level of pubic symphysis DNC is known to have contact with cavernous nerves^38^ that are exclusively autonomic^17^ which might be contributing differences in fiber counts. Even tough DNC itself contains autonomic fibers; it is mostly somatic due to the vast majority of sensory fibers that innervate corpuscular and free nerve endings^39^. One limitation of this study is that we didn`t differentiate between the subtypes of fibers yet we can speculate that most of the fibers we counted are somatic fibers according to previous research. Further studies are required to understand the composition of DNC in female pleasure.

Our study provides a quantitative mapping of clitoral innervation and structure. Quantitative information indicates that while the absolute number of nerve fibers is ~ 60% lower in the clitoris, albeit with a higher proportion of myelinated fibers, the innervation density is markedly higher than in the penis. These data will inform not only our understanding of differences and similarities between female and male genitals and their sensory function but also reveal some of the neglected aspects of female sexuality. In the scientific literature glans penis got 20 times more attention than glans clitoris, which subtly means we know less about clitoris^40^. We require further clinical and basic research to understand the healthy female functioning, so dysfunctions can be addressed and treated. Many sexual dysfunctions such as clitoral and vulvar pain, arousal and orgasm disorders still do not get the attention that premature ejaculation or erectile dysfunctions get. In parallel, heteronormative sexual narratives that emphasize penile-vaginal intercourse shaped the definition of the female pleasure. Consequently, an “orgasm gap” occurred in which women have less orgasm and sexual pleasure during heterosexual relationships^41^. Thus, every piece of information contributing to the comprehension of female pleasure and sexual health holds a value in order to close the orgasm gap.

## Supplementary Information

Below is the link to the electronic supplementary material.

Supplementary Material 1

## Data Availability

The data that support the findings of this study are available from the corresponding author upon reasonable request.
