# Supplementary material for: Innervation pattern and fiber counts of the human dorsal nerve of clitoris
Source: Sci Rep. 2024 Oct 4;14:23060. doi: 10.1038/s41598-024-72898-8 (PMC11452485; doi:10.1038/s41598-024-72898-8)
Supplement: Supplementary file 1 — Supplementary Material 1 [file 41598_2024_72898_MOESM1_ESM.docx]

**Supplementary Tables**

**Table 1. Azan Trichrome Staining protocol**

| **Step** | **Reagent** | **Duration** |
| --- | --- | --- |
| 1 | Xylol | 5 min |
| 2 | Xylol | 5 min |
| 3 | 100 % ethanol | 3 min |
| 4 | 100% ethanol | 3 min |
| 5 | 96 % ethanol | 1 min |
| 6 | Aniline alcohol | 5 min |
| 7 | Distilled water | 10 sec |
| 8 | Azocarmine | 55 min at 55- 58 °C |
| 9 | Tap water | 10 sec |
| 10 | Aniline alcohol | 5 min |
| 11 | Acetic acid in ethanol 1% | 1 min |
| 12 | Tap water | 10 sec |
| 13 | Phosphotungstic acid 5% | 2 hours |
| 14 | Tap water | 10 sec |
| 15 | Aniline blue- Orange G | 20 min |
| 16 | Tap water | 10 sec |
| 17 | 96 % ethanol | 1 min |
| 18 | 100% ethanol | 2 min |
| 19 | Xylol | 5 min |
| 20 | Xylol | 5 min |
| 21 | Eukitt |  |

| **Table 2. Comparison of clitoris and penis measurements**  **Clitoris Measurements** | | | | | |
| --- | --- | --- | --- | --- | --- |
| **Donor** | **Mean Circumference (mm)** | **Length (mm)** | **Surface Area (mm^2^)** | **Total Crura Fiber Counts** | **Innervation Density** |
| 1 | 38 | 37 | 1408 | 6742 | 4,8 |
| 2 | 43 | 38 | 1616 | 4657 | 2,9 |
| 3 | 36 | 27 | 964 | 5572 | 5,8 |
| **Mean** | **39** | **34** | **1330** | **5657** | **4** |
|  |  |  |  |  |  |
|  |  |  |  |  |  |
| **Penis Measurements** | | | | | |
| **Donor** | **Mean Circumference (mm)** | **Length (mm)** | **Surface Area (mm^2^)** | **Total Root Fiber Counts** | **Innervation Density** |
| 1 | 111 | 119 | 13220 | 14048 | 1,1 |
| 2 | 102 | 128 | 13036 | 7572 | 0,6 |
| 3 | 100 | 113 | 11292 | 6254 | 0,6 |
| 4 | 105 | 120 | 12632 | 7240 | 0,6 |
| **Mean** | **104** | **120** | **12545** | **8779** | **0,7** |
|  |  |  |  |  |  |

**Supplementary Figure 1**

**
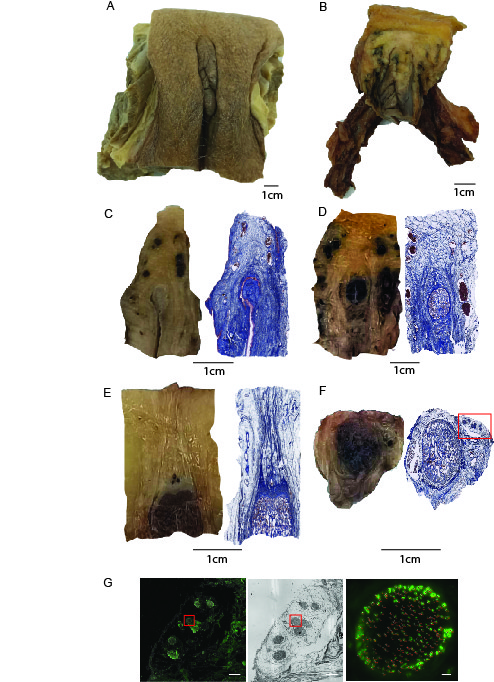
**

**Supplementary Figure Legends**

**Figure 1. Detailed depiction of experimental**

**(a)** Another vulva is shown in frontal view.

**(b)** Vulva is skinned and isolated from the connective tissue and vestibular bulbs. Bilateral crura and the body of the clitoris are shown in frontal view. Distally, the tip of glans clitoris is covered by prepuce while proximally fatty tissue dorsal to DNC bundles are shown.

**(c)** Cross section of distal clitoris. Left panel shows glans clitoris together with big blood vessels. Right panel shows the same section with trichrome azan. Scale bar is adjusted according to left panel.

**(d)** Cross section shows the middle of clitoral body. Left panel shows corpora cavernosa situated dorsal to corpus spongiosum. Blood vessels are stained red. Scale bar is adjusted according to left panel.

**(e)** Cross section shows proximal clitoris. Left panel shows corpora cavernosa situated ventral to three deep blood vessels. DNC bundles are located dorsal to the blood vessel. On the right panel, connective tissue that encapsulates bundles is observed in web-like formation. Scale bar is adjusted according to left panel.

**(f)** Cross section of crus shows the corpus cavernosum. Neurovascularbundle is encapsulated by connective tissue and situated between corpus cavernosum and ischiopubic rami. On the left panel this location is upper right corner. This observation is further confirmed with trichrome azan staining. Web-like tissue surrounding bundles are highlighted. Scale bar is adjusted according to left panel.

**(g)** The consecutive slides show the area highlighted on panel F. Left panel shows the NVB bundles in neurofilament H while middle panel in luxol fast blue. Right panel shows our manual counting process. NfH positive cells
